# Human Lacrimal Gland Derived Mesenchymal Stem Cells – Isolation, Propagation, and Characterization

**DOI:** 10.1167/iovs.64.10.12

**Published:** 2023-07-13

**Authors:** Jilu Jaffet, Aparna Mohanty, Induvahi Veernala, Swati Singh, Mohammad Javed Ali, Sayan Basu, Geeta K. Vemuganti, Vivek Singh

**Affiliations:** 1 Centre for Ocular Regeneration, Professor Brien Holden Eye Research Centre, Champalimaud Translational Centre for Eye Research, L. V. Prasad Eye Institute, Hyderabad, Telangana, India; 2 Manipal Academy of Higher Education (MAHE), Manipal, Karnataka, India; 3 School of Medical Sciences, University of Hyderabad, Gachibowli, Hyderabad, Telangana, India; 4 Govindram Seksaria Institute of Dacryology, L. V. Prasad Eye Institute, Hyderabad, Telangana, India

**Keywords:** lacrimal gland-mesenchymal stem cells (LG-MSCs), dry eye disease (DED), mesenchymal stem cells (MSCs), lacrimal gland (LG), stem cell therapy, tear film

## Abstract

**Purpose:**

The existing treatment options for dry eye disease (DED) due to lacrimal gland (LG) dysfunction are mainly palliative. Mesenchymal stem cells (MSCs) based therapies and 3D-LG organoids have been explored as a curative option for LG regeneration in animal models. Human LG epithelial cultures are previously established and, here, we aim to isolate and characterize the spindle-shaped cells obtained from primary human LG cultures in order to unveil its MSC property.

**Methods:**

Normal human lacrimal glands were obtained from individuals undergoing LG debulking surgery. The conditions for human LG-MSC culture were standardized to obtain pure population of LG-MSCs at passage 3. Population doubling time (PDT), expression of phenotypic markers, tri-lineage differentiation, colony forming potential, and gene expression analysis were carried out to assess the phenotypic and genotypic characteristics compared to bone marrow-MSCs (BM-MSCs).

**Results:**

Our data show that these spindle-shaped cells exhibit similar phenotypic expression, colony-forming ability, and trilineage differentiation like BM-MSCs. Moreover, the gene expression also did not show any significant difference, except for increased IL1-β in LG-MSCs. The LG-MSCs do not express any lacrimal epithelial markers unlike LG tissue.

**Conclusions:**

This study reveals the first-time evidence for the presence of MSC population within the human LGs, and these cells might play a role in maintaining healthy microenvironment within normal LG and repair in diseased LGs.

Lacrimal functional unit (LFU) is the active component maintaining the ocular surface homeostasis. LFU is a multifunctional arm that comprises of the main and accessory lacrimal glands (LGs), the ocular surface (the cornea, the meibomian glands, and the conjunctiva) and the sensory and the motor nerves that interconnects them.^1^ Maintaining a healthy tear film is inevitable for preserving the transparency of the cornea and ensuring the clear vision.^2^ Alterations in the tear film composition can be due to the abnormal functioning of lacrimal glands, meibomian glands, or the conjunctival goblet cells.^3^ Any abnormality in the normal functioning of these glands can destabilize the tear film homeostasis, and causes dryness in the ocular surface, leading to a chronic condition known as dry eye disease (DED).^4^ Sustained usage of artificial tear drops and lubricants are the only existing DED management option. In this study, we restrict our focus on DED caused due to LG dysfunction and hence characterizing the stem cell population within human LGs.

The LG is a bilobed exocrine gland located in the superotemporal orbit and is separated into two lobes by the aponeurosis of levator palpebrae superioris.^5^^,^^6^ The orbital lobe is comparatively bigger and is located within the lacrimal fossa whereas the smaller palpebral lobe is found continuous with the upper eye lid. The aqueous component reaches the ocular surface through the secretory ducts from the palpebral lobe of lacrimal gland. The acinar epithelial cells are the major secretory cells of the lacrimal gland and it comprises 80% of the total LG.^4^

Understanding the structure and the cell types of LGs are important in investigating the LG-associated disorders and assessing its functionality. Initially, LG cultures were established with the aim of restoring LG function in patients with DED. This could be achieved either by generating transplantable 3D LG organoids or with the help of stem cells capable of LG regeneration. In severe dry eye cases with no aqueous production due to loss of LG function, cell therapy using LG stem cells or LG organoids could be the promising approach.^4^ Earlier, our group has reported the establishment of human LG epithelial cultures with secretory function and provided preliminary evidence for the presence of epithelial stem cells within human LGs.^7^ Apart from the 2D LG epithelial cultures, 3D floating lacrispheres were also established from the cultured human LG epithelial cells and the stem cells.^8^ Other than the epithelial population, LG stroma is known to have various other cell types like fibroblasts, mast cells, etc.^9^ Some studies in rodents have shown the presence of mesenchymal stem cells (MSCs) within their LGs^10^^,^^11^ and those cells were reported to have a role in LG regeneration in DED models.^12^^,^^13^ The combined use of epithelial cells along with the MSCs could be useful in generating 3D LG organoids for transplantation in DED models. There are no previous reports investigating the presence of MSCs within human LGs. During explant culture of human LGs, we observed the growth of spindle shaped cells along with the epithelial sheets. Here, we aim to investigate whether these primary cultures, that is, the spindle shaped cells isolated from human lacrimal glands were of mesenchymal origin by comparing them with human bone marrow derived MSCs (BM-MSCs) in terms of plasticity and immunophenotype.

BM-MSCs are the heterogenous population of multipotent stem cells having high plasticity, potential to differentiate into mesodermal lineage and high regenerative potential.^14^ BM-MSCs show positivity for CD73, CD90, and CD105 and does not express CD45, CD34, and HLA-DR meeting the criteria of MSCs as per the International Society for Cellular Therapy (ISCT) definition.^15^ Apart from bone marrow,^16^ MSCs have also been identified in multiple tissues namely adipose tissues,^17^^,^^18^ dental tissues,^19^^,^^20^ Wharton's jelly,^21^^,^^22^ limbus,^23^^,^^24^ placenta,^25^ salivary gland,^26^ synovial fluid,^27^ umbilical cord,^28^ peripheral blood,^29^ etc. Moreover, our group has also established and characterized MSCs from rat bone marrow,^30^ human bone marrow,^31^ and limbus.^23^^,^^32^ However, over the decades, BM-MSCs have served as the gold standard for comparison of MSCs from other sources. Hence, we focused on the spindle shaped cells obtained from human LG cultures and compared them with primary human BM-MSCs for their characteristics with respect to culture characteristics, phenotype, trilineage differentiation, and the gene expression. From the findings of this study, we speculate that we would provide practical value and prospective research ideas for future clinical applications in regenerative medicine.

## Methods

This research study was approved by the institutional review board of L. V. Prasad Eye Institute (Ethics Ref. No. LEC-BHR-P-04-21-622), University of Hyderabad ethical committee (Ref. No. UH/IEC/2022/245), the Institutional Committee for Stem Cell Research of L. V. Prasad Eye Institute (IC SCR Ref. No. 06-21-004), University of Hyderabad Stem Cell Research Committee (Ref. No. UH/ICSCR/2022/2), and followed the tenets of Declaration of Helsinki, including the informed consent from all subjects. All the experiments in methodology were at least performed in three different human lacrimal gland tissues and were performed in triplicates (Human LG donor 1-11/M, LG donor 2-40/M, and LG donor 3-64/F).

### Isolation and Establishment of Cultures

Normal human LGs were harvested from individuals undergoing LG debulking surgery for therapeutic indications. After diagnostic procedures, the leftover sample of the LG tissue was collected in DMEM F-12 (D0547; Sigma Aldrich, USA) media supplemented with 2% fetal bovine serum (FBS; 16000036;, Thermo Fisher, USA) in ice. The tissue was rinsed with 1X phosphate buffered saline (PBS; 14190250; Thermo Fisher, USA) followed by 2X antibiotic-antimycotic (15240062; Thermo Fisher, USA) twice. The tissue was minced into smaller parts using a #15 sterile scalpel blade. The minced tissues were used for establishing explant cultures. Briefly, the minced tissues were put in a T25 flask and placed in the incubator for 1 minute with less than 1 mL of media for better attachment. Upon attachment, complete media (DMEM F-12 media with 5 µg/mL insulin (12585014; Thermo Fisher, USA), 10 ng/mL EGF (PHG0311L; Thermo Fisher, USA), 1% [vol/vol] antibiotic-antimycotic, 2 mM L-Glutamine and 10% FBS was added to the flask, left undisturbed for at least 3 days, followed by media change every alternate day. The explants were cultured for 14 days, the mixed population of cells were passaged and seeded in a T25 flask. The cells were passaged until a pure population of spindle shaped cells were obtained (P3), which was used for further characterization.

For comparison of BM-MSCs with LG-stromal cells, BM-MSCs were isolated from human leftover bone marrow aspirate samples after diagnostic procedures by Histopaque -1077 (10771; Sigma Aldrich, USA) gradient-based centrifugation method. Briefly, human bone marrow aspirate was collected from leftover bone marrow tap after diagnostic procedures. Mononuclear cells were obtained using histopaque gradient at 400 g for 30 minutes. The buffy coat containing mononuclear cells was carefully collected and washed with 1X PBS twice by centrifuging at 250 g for 10 minutes. The mononuclear cells were resuspended in Dulbecco’s Modified Eagle’s Medium (1190565; Thermo Fisher, USA), supplemented with 10% FBS, and seeded in T25 flask. The flask was left undisturbed for 48 hours. Non-adherent cells were discarded, and fresh media was added to the flask. Upon reaching confluency, the cells were passaged using TrypLE (12604013; Thermo Fisher, USA).

### Colony Forming Unit Assay

Colony forming unit (CFU) assay was performed to determine the ability of BM-MSCs and LG-stromal cells to grow into colonies from single cells. Both the cell types were plated in T25 tissue culture flasks at a density of 250 cells/cm^2^. After 14 days, the cultures were fixed in ice-cold methanol for 10 minutes at 4°C and stained with 3% crystal violet for 10 minutes. The colony count was calculated and colonies with less than 2 mm in diameter or faintly stained were excluded.

### Population Doubling Time

For BM-MSCs and LG-stromal cells, population doublings were calculated from passages 3 to 6. The cells were seeded at a density of 5000 cells/cm^2^ at each passage and trypsinized upon confluency. The population doublings were calculated as follows:

$$\begin{array}{c} Number\;of\;Cell\;Doublings\;\left(NCD\right)=\frac{Log\;10\left(\frac{y}{x}\right)}{log10^{2}} \end{array}$$

Where “y” is the final density of the cells and ‘x’ is the initial seeding density of the cells.

### Characterization of LG-Stromal Cells and BM-MSCs

#### Flow Cytometry

LG-stromal cells and BM-MSCs were characterized for panel of mesenchymal markers CD90 (1:100, B36121; Beckman Coulter, USA), CD105 (1:100, B76299; Beckman Coulter, USA), CD73 (1:100, B68176; Beckman Coulter, USA), hematopoietic CD34 (1:100, IM1870; Beckman Coulter, USA), CD45 (1:100, A07783; Beckman Coulter, USA), and HLA-DR (1:100, B36291; Beckman Coulter, USA) using fluorescent-activated cell sorting (FACS). Briefly, a single cell suspension (0.1 × 10^6^ cells each) of LG-stromal cells and BM-MSC at passage 3, were harvested by centrifuging at 400 g for 5 minutes. The cells were further washed twice with 1X PBS at 400 g for 5 minutes. Then the cells were incubated with saturating concentrations of respective conjugated primary antibodies in 2% FBS, for 20 minutes in the dark at room temperature (RT). After 2 washes, the cells were centrifuged at 400 g for 5 minutes, resuspended in PBS, and then analyzed. Cell fluorescence was evaluated by flow cytometry in FACS CytoFlex S cell analyzer (Beckman Coulter, Indianapolis, IN, USA) and data were analyzed using CytExpert software (Beckman Coulter, Indianapolis, IN, USA). A total of 10,000 events were acquired to determine the positivity of different cell surface markers used. Unstained cells of BM-MSCs and LG-stromal cells were used as the negative control. Respective isotype controls were also used for excluding the nonspecific binding (Supplementary Fig. S3).

#### Immunofluorescence

The expression of designated markers, CD73 (1:200, 13160; Cell Signalling Technology, Danvers, MA, USA), CD90 (1:200, ab181469; Abcam, UK), CD105 (1:200, sc-376381; Santa Cruz Biotechnology, USA), CD45 (1:200, 13197; Cell Signalling Technology, USA), HLA-DR (1:200, ab55152; Abcam, UK) and Vimentin (1:200, sc-6260; Santa Cruz Biotechnology) was additionally confirmed by immunofluorescence. LG-stromal cells and BM-MSCs at passage 3 were seeded on coverslips in 12-well plates and cultured until 70% confluency. Cells were then fixed with 4% paraformaldehyde for 10 minutes at RT. The cells were then incubated in 0.25% Triton X-100 for 5 minutes. Nonspecific reactions were blocked with 2.5% BSA in 1X PBS for 45 minutes at RT. After 2 washes, cells were then incubated with primary antibodies overnight at 4°C, which was detected using Alexa fluor 594-conjugated secondary antibody (1:400, anti-rabbit, A11012; Thermo Fisher, USA and 1:400, anti-mouse, A11005; Thermo Fisher, USA). Negative controls were cells incubated only with the respective secondary antibody. Cells were mounted with Fluoroshield mounting medium with DAPI (ab104139; Abcam, UK). The stained preparations were visualized with a fluorescence microscope (Axio Scope A1; Carl Zeiss AG, Oberkochen, Germany) and images were captured.

For LG tissues, the tissues were fixed in 10% buffered formalin and 4 µm thin sections were prepared from paraffin embedded tissue blocks. The tissue sections were heated at 70°C for 7 minutes for de-paraffinization. These sections were dipped in an exchange of xylene thrice for 5 minutes each and rehydration was performed by dipping in 100%, 90%, and 80% alcohol, respectively, for 5 minutes each in a rocker. Slides were rinsed in running tap water for 1 minute. Antigen retrieval was performed by heating the slides in citrate buffer (pH = 6) thrice for 5 minutes each in a microwave. Then, the slides were allowed to cool down to RT and staining protocol was continued as described previously. Tissue sections were stained using ABCB5 (1:500, ab140667; Abcam, UK), C-Kit (1:200, SC393910; Santa Cruz Biotechnology, USA), Lysozyme (1:250, ab108508; Abcam, UK), Pan-CK (1:500, ITM0192; G-Biosciences, USA), and Ki67 (1:250, ab16667; Abcam, UK).

### Tri-Lineage Differentiation

The potential of both the cell types to undergo trilineage differentiation was evaluated by culturing in MesenCult Osteogenic differentiation media (05465), adipogenic differentiation medium (05412), and chondrogenic differentiation media (05455). In brief, both the cell types were seeded in 24-well plates at a density of 5000 cells/ cm^2^ and cultured in a complete medium until confluency. The medium in the culture was then replaced with respective MesenCult differentiation media and incubated for 21 days. Subsequently, the cultures were fixed in 4% paraformaldehyde for 10 minutes and washed twice with 1X PBS. For osteogenic differentiation, fresh 2% Alizarin Red S staining was performed for 3 to 5 minutes and later washed twice with distilled water. Fixed cells were stained with fresh 0.3% Oil Red-O solution for 10 minutes for adipogenic differentiation. The chondrogenic differentiation was identified by staining with 1% Alcian blue in 3% acetic acid solution (pH 2.5).^33^^,^^34^ To quantify the extent of differentiation, the stains were eluted from the cells using the protocols previously described.^35^^–^^37^ The intensity was measured using a UV-Vis spectrophotometer (SpectraMax M3; Molecular Devices, San Jose, CA, USA).

### Gene Expression Analysis

P3 cells of BM-MSCs and LG-stromal cells were resuspended in RNAiso Plus (91089109; TAKARA) and RNA isolation was performed using the manufacturer's instructions. The RNA was quantified using Nanodrop (ND-2000C; Thermo Scientific, USA) and a total of 2 µg RNA was used for cDNA synthesis. The cDNA was synthesized using SuperScript III First-Strand Synthesis System (18080051; Invitrogen, USA) according to manufacturer's protocol. The real-time PCR was performed using DyNAmo ColorFlash SYBR Green qPCR Kit (F416L; Thermo Scientific, USA). The primers involved are listed in the Table. Expression of all the below mentioned markers was normalized using β-actin as the internal control. The expression of each gene in LG-stromal cells were calculated as a relative fold change with respect to the expression in BM-MSCs. The reaction mix without the respective cDNA was used as the negative control in all PCRs.

**Table.** List of Primers Used For Gene Expression Analysis

Primer	Sequence	Product Size
Βactin	FP: TCTACAATGAGCTGCGTGTG	314 bp
	RP: GGTGAGGATCTTCATGAGGT	
CD105	FP: CGGTGGTCAATATCCTGTCGAG	109 bp
	RP: AGGAAGTGTGGGCTGAGGTAGA	
CD73	FP: GGCTGCTGTATTGCCCTTTG	175 bp
	RP: TACTCTGTCTCCAGGTTTTCGG	
CD90	FP: AGCATCGCTCTCCTGCTAAC	230 bp
	RP: CTGGTGAAGTTGGTTCGGGA	
SOX9	FP: GGCAAGCTCTGGAGACTTCTG	138 bp
	RP: CCCGTTCTTCACCGACTTCC	
RUNX2	FP: CCACTGAACCAAAAAGAAATCCC	129 bp
	RP: GAAAACAACACATAGCCAAACGC	
CD45	FP: CTTCAGTGGTCCCATTGTGGTG	107 bp
	RP: CCACTTTGTTCTCGGCTTCCAG	
CD34	FP: CCTCAGTGTCTACTGCTGGTCT	144 bp
	RP: GGAATAGCTCTGGTGGCTTGCA	
IL-1β	FP: GCAAGGGCTTCAGGCAGGCCGCG	96 bp
	RP: GGTCATTCTCCTGGAAGGTCTGTGGGC	
Lysozyme	FP: ACTACAATGCTGGAGACAGAAGC	157 bp
	RP: GCACAAGCTACAGCATCAGCGA	
Vimentin	FP: GGGACCTCTACGAGGAGGAG	200 bp
	RP: CGCATTGTCAACATCCTGTC	
MMP9	FP: GATGCGTGGAGAGTCGAAAT	338 bp
	RP: CACCAAACTGGATGACGATG	
TGF-β1	FP: GAGGTGACCTGGCCACCATTCAT	194 bp
	RP: TCCGCAAGGACCTCGGCTGG	
HIF-1α	FP: CAAGAACCTACTGCTAATGC	125 bp
	RP: TTATGTATGTGGGTAGGAGATG	
Aquaporin V	FP: AGCTGATTCTGACCTTCCAGC	222 bp
	RP: CTACCCAGAAAACCCAGTGAGC	
Lactoferrin	FP: CTCCAGACCGCAGACATGAA	630 bp
	RP: CTGGGAGGAGAAGGCACATT	
Lacritin	FP: AGATGCCTCCTCTGACTCGACG	131 bp
	RP: AAGTCTCCTGGGCTGTTGTGGT	
ABCG2	FP: GTTCTCAGCAGCTCTTCGGCTT	399 bp
	RP: TCCTCCAGACACACCACGGATA	

### Statistical Analysis

The mean and standard deviation were calculated in Microsoft Excel and the graphs were plotted using GraphPad Prism 9 (GraphPad Software, San Diego, CA, USA). Statistical significance was analyzed using the Student's *t-*test for nonparametric data. The data is presented as mean values ± SD, obtained from at least three independent experiments performed. Any *P* ≤ 0.033, *P* ≤ 0.002, and *P* ≤ 0.0002 were considered to be significant and are represented by *, **, and ***, respectively, whereas *P* > 0.033 meant nonsignificant and are represented as “ns.”

## Results

### Isolation and Establishment of Cultures

**Figure 1.**
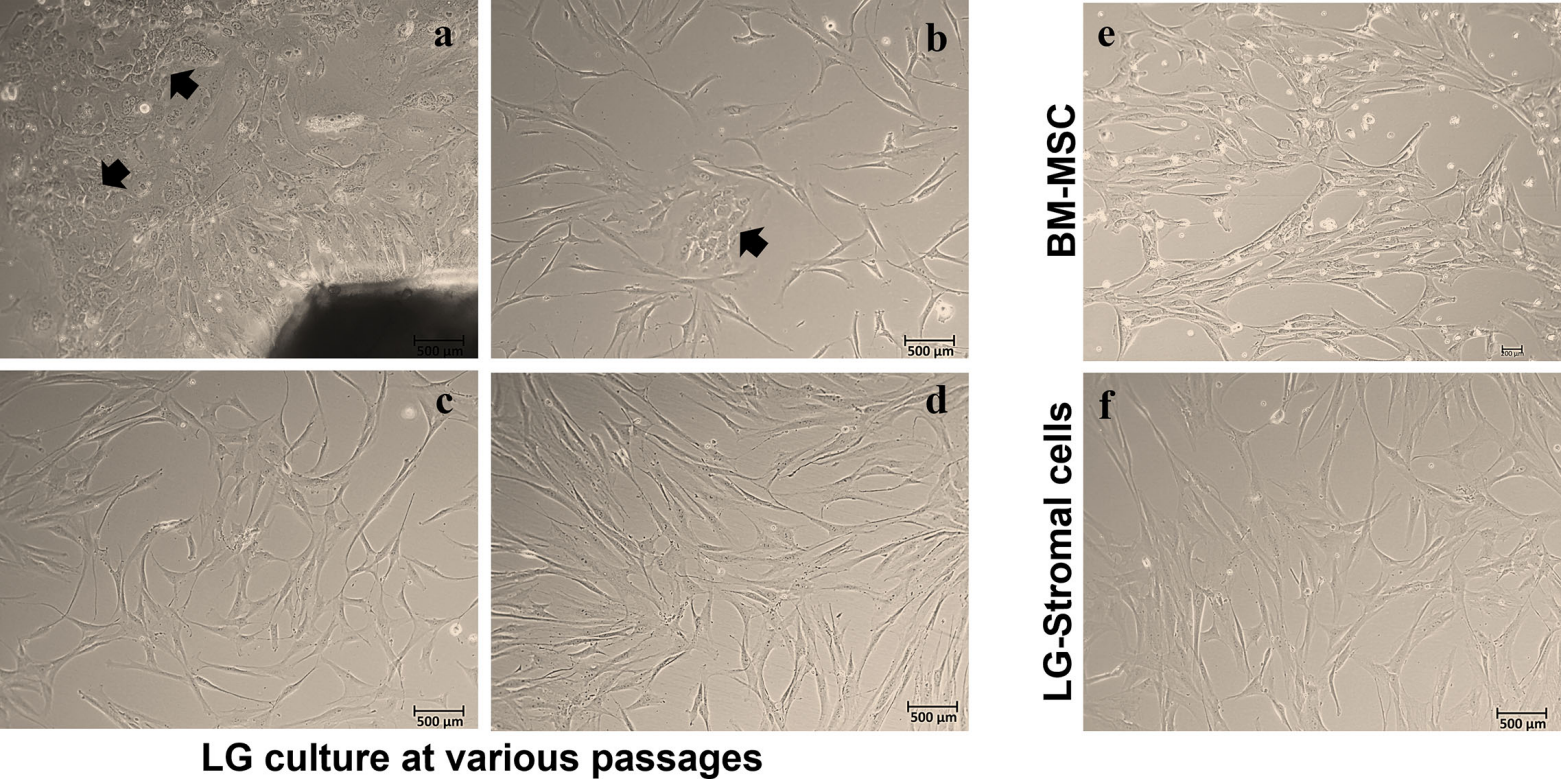
Isolation and establishment of human lacrimal gland stromal stem cells at 10X objective magnification. (**A**) The growth of mixed population of cells from the human lacrimal gland explant. (**B**) A mixed population of epithelial like sheets and spindle shaped cells seen in passage 1 (*black arrows* indicate the epithelial like sheets in early passages). (**C, D**) Spindle-shaped fibroblast-like cells were predominantly observed in passages 2 and 3, respectively. (**E, F**) BM-MSCs and LG-Stromal stem cells show similar spindle shaped morphology.

Explant culture of LGs showed mixed population of cells (Fig. 1A) with polygonal (black arrow) as well as spindle shaped cells. Mixed population of cells (Fig. 1B) were observed until passage 2 (Fig. 1C). Pure population of spindle shaped cells with fibroblast like morphology were obtained at passage 3 (Fig. 1D). The cells obtained at passage 3 were the first pure population of spindle shaped cells (LG-stromal stem cells). The P3 BM-MSCs (Fig. 1E) and the LG-stromal cells (Fig. 1F) were similar in their fibroblastic morphology and its spindle shape. Human LG-stromal cells were also passaged up to P15 and cells maintained its spindle shaped morphology (data not shown). However, a study is ongoing to know the Hayflick limit as well as the number of passages these cells can be cultured maintaining its MSC properties for clinical uses.

### Hematoxylin and Eosin Staining of Human LGs

**Figure 2.**
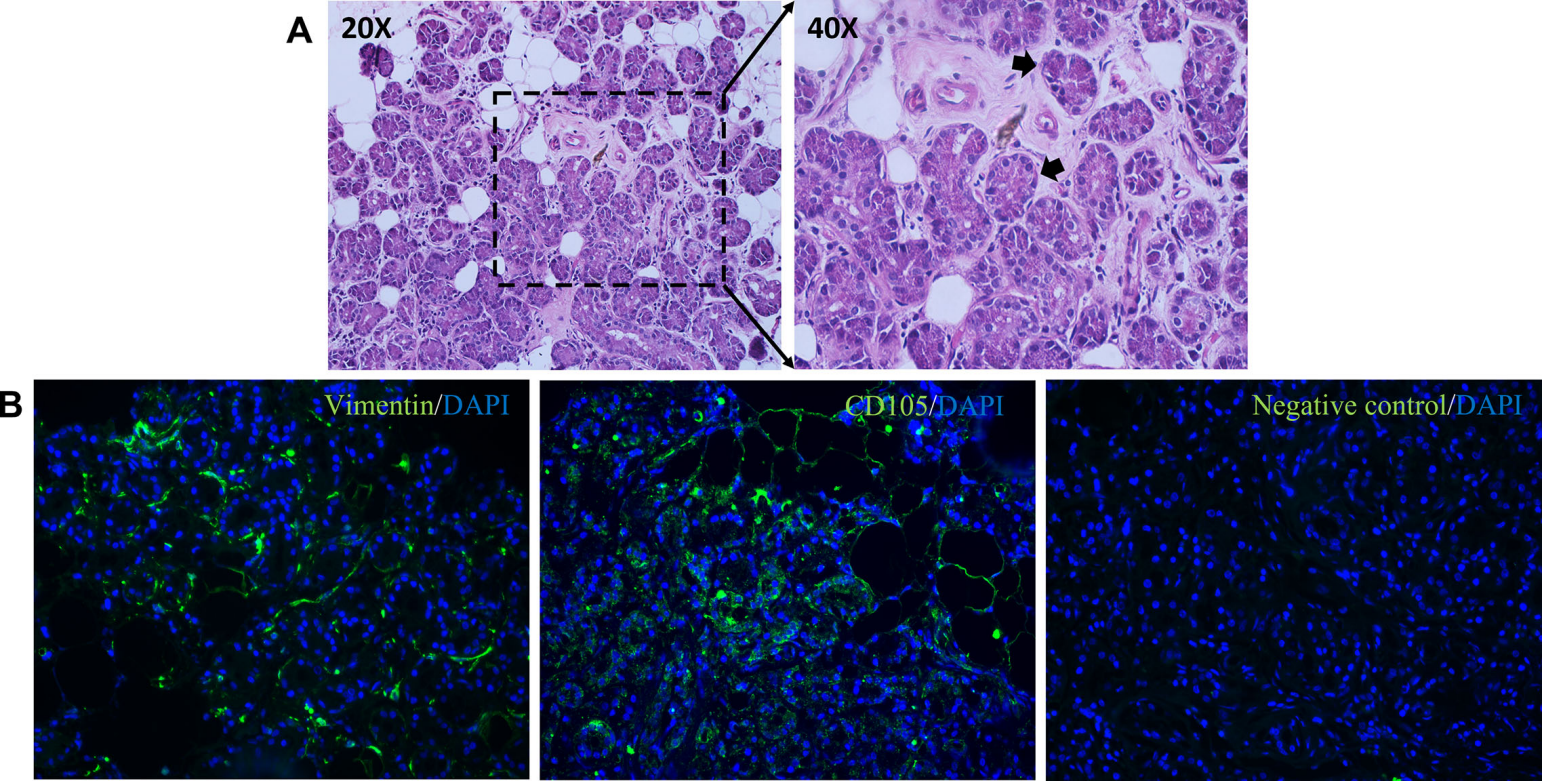
(**A**) Sections of human lacrimal gland tissue processed for Hematoxylin and Eosin stain imaged at 20X and 40X magnifications. *Black arrows* indicating the round structures known as acini within the LG tissue. (**B**) The human LG tissues expressing MSC markers Vimentin and CD105. Images were captured at 40X magnification.

The hematoxylin and eosin (H&E) staining of human LGs show round acini, which comprises the secretory acinar cells of the LG. The LGs did not show any signs of severe inflammation and tissue damage (Fig. 2A). The expression of MSC specific markers, Vimentin and CD105, were observed in human LG tissues. Vimentin and CD105 expression were localized surrounding the acinar epithelial cells of the LGs (Fig. 2B).

### Colony Forming Unit and Population Doublings

**Figure 3.**
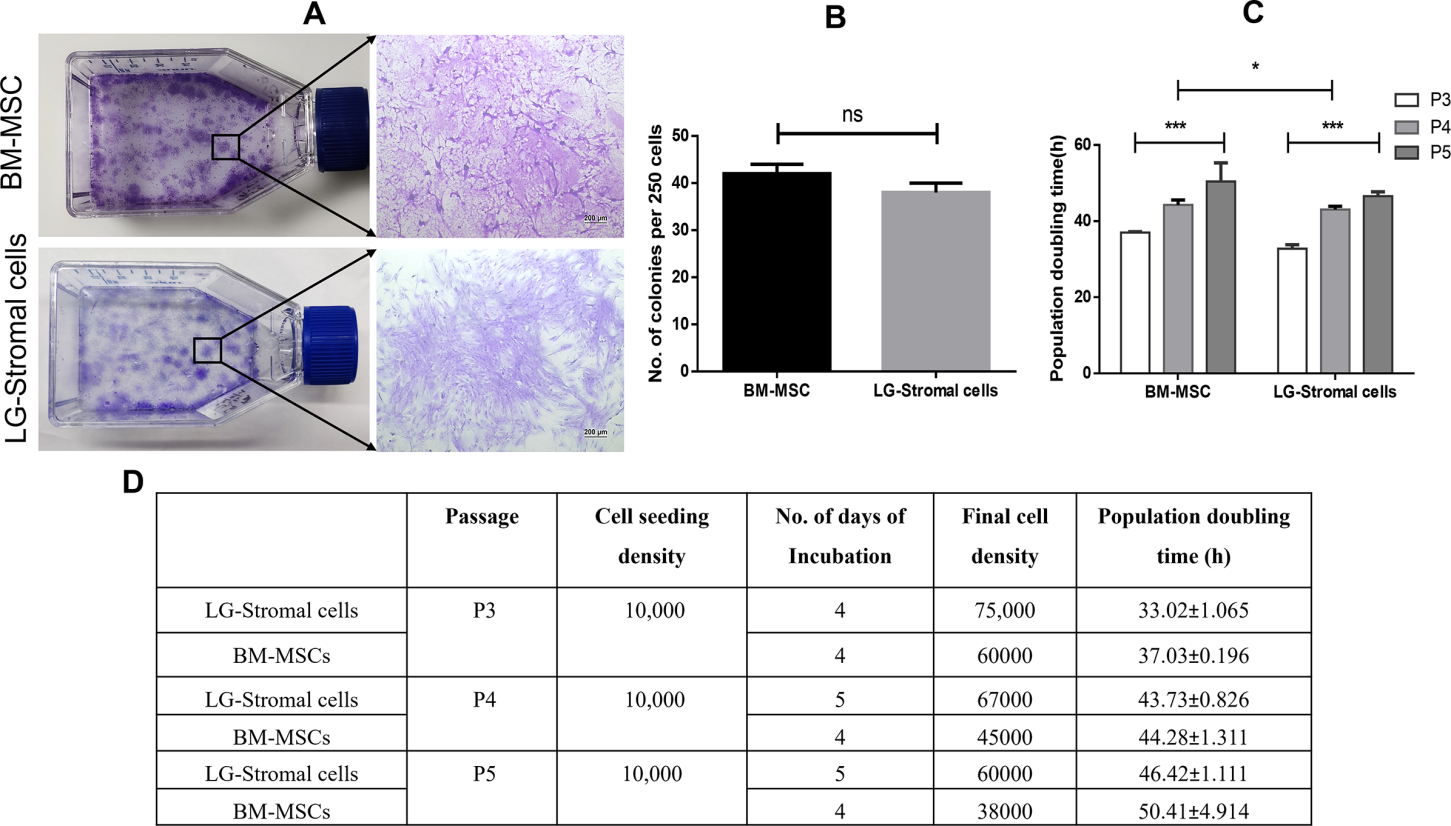
(**A**) The colony-forming efficiency of both BM-MSCs and LG-stromal cells were 16.13 ± 0.61% and 15.0 ± 1.0%, respectively. Colonies greater than 2 mm were shown as the magnified image (Scale bar = 200 µm). (**B**) The number of colonies formed upon seeding 250 cells were 40.3 ± 1.5 and 37.6 ± 2.5 for BM-MSCs and LG-stromal cells, respectively. (**C**) Population doubling time of BM-MSCs and LG-stromal cells was measured at each passage from passage 3 until passage 5. * *P* ≤ 0.033, and *** *P* ≤ 0.0002. (**D**) The Table denotes the initial seeding density and final cell density along with the calculated populated doublings.

When LG-stromal cells and BM-MSCs were seeded at a density of 250 cells/T25 flask, the number of colonies formed were found to be 37.6 ± 2.5 and 40.3 ± 1.5, respectively (Figs. 3A, 3B). In addition, the CFU efficiency was calculated to be 15.0 ± 1.0% and 16.13 ± 0.61% respectively for LG-Stromal cells and BM-MSCs. Both the cell types were plated at a density of 5000 cells/cm^2^ at different passages and the doubling time increased progressively (Fig. 3C). The population doubling time was found to be significantly increasing in both LG-stromal cells as well as BM-MSCs. Results are summarized in the Figure 3D from passage 3 to passage 5.

### Flow Cytometry

**Figure 4.**
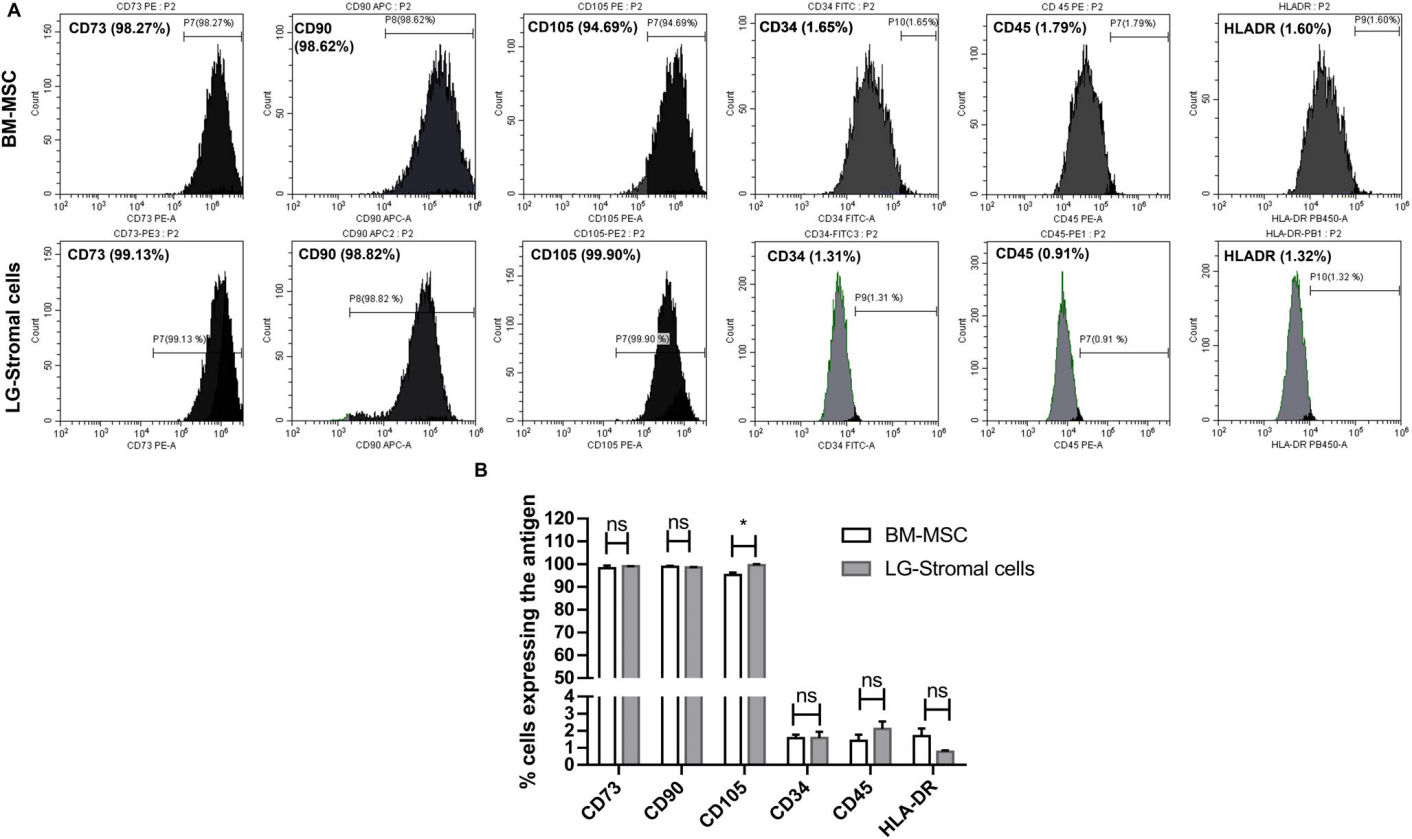
Flow cytometric analysis of cell surface antigens was performed using BM-MSCs and LG-stromal cells in the P3 and the positive expression of CD73 (*P* = 0.25), CD90 (*P* = 0.42), and CD105 (*P* = 0.003) was detected, whereas lacking expression of negative markers CD34 (*P* = 0.95), CD45 (*P* = 0.09), and HLA-DR (*P* = 0.02).

According to the International Society for Cell & Gene Therapy (ISCT) criteria, MSCs must express (>95%) surface markers CD73, CD90, and CD105 and lack the expression (<3%) of CD34, CD45, and HLA-DR. The FACS analysis of our study was in accordance with the criteria proposed by ISCT. Both LG-stromal cells and BM-MSCs showed similar expression patterns and expressed surface markers CD73 (99.14 ± 0.04% and 98.2 ± 1.22%), CD90 (98.59 ± 0.23% and 98.86 ± 0.48%), CD105 (99.61 ± 0.40% and 95.24 ±1 0.11%), and were negative for CD34 (1.57 ± 0.36% and 1.56 ± 0.21%), CD45 (2.11 ± 0.42% and 1.41 ± 0.35%), and HLA-DR (0.77 ± 0.06% and 1.68 ± 0.44%) (Figs. 4A, 4B).

### Immunofluorescence

**Figure 5.**
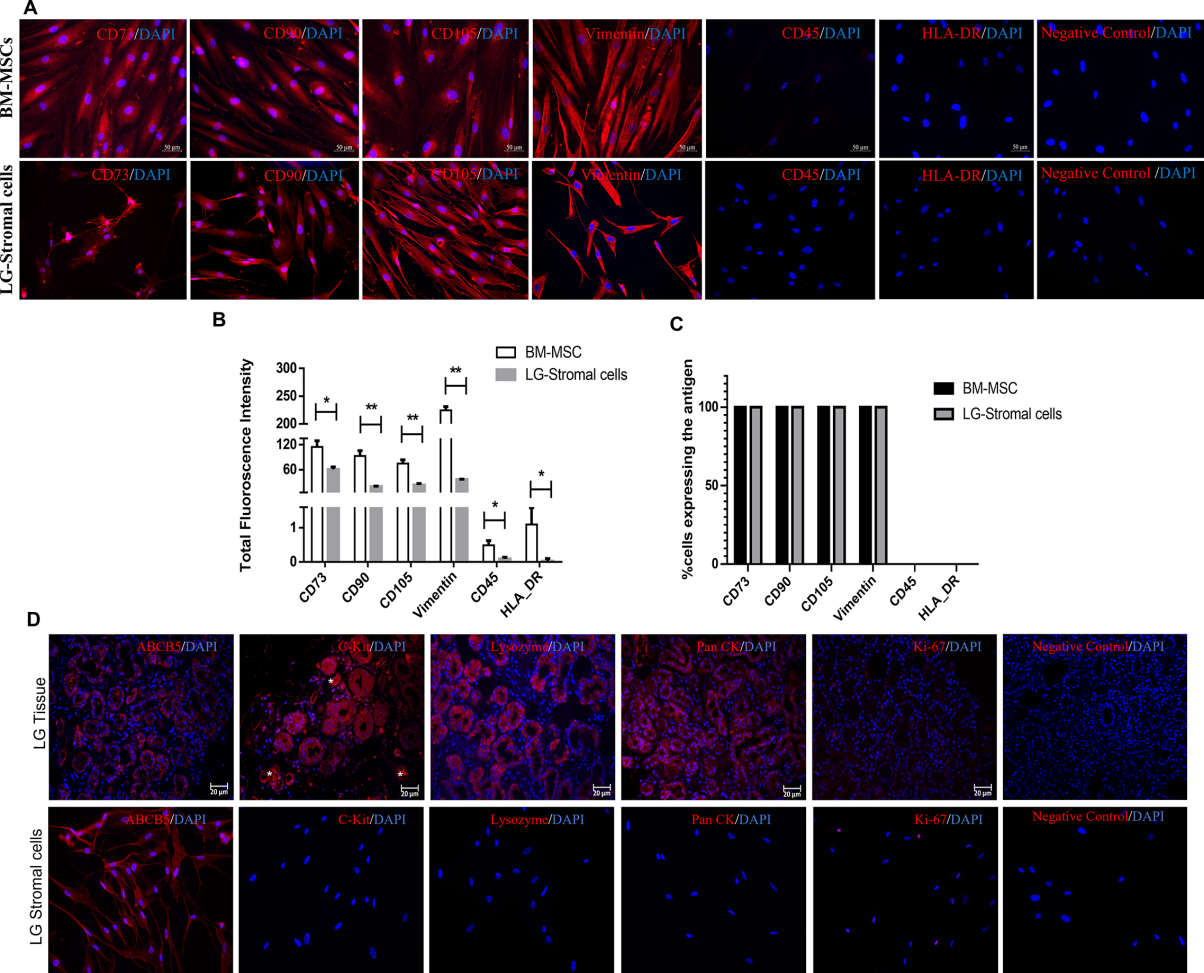
Immunophenotypic analysis of BM-MSCs and LG-stromal cells. (**A**) MSCs showed positive expression for CD73, CD90, CD105, and Vimentin, and negative for CD45 and HLA-DR with Alexa fluor 594 conjugated secondary antibody. The nucleus was counterstained with DAPI. Images were captured at 40X magnification. (**B**) Quantitative analysis of the expression of various markers of BM-MSCs and LG- stromal cells in terms of corrected total cell fluorescence intensity. Data are shown as absorbance mean ± standard deviation from three separate experiments. (**C**) The quantitative analysis of the percentage of cells expressing various markers per area. Representative data of three areas taken for quantification. * *P* ≤ 0.033, and ** *P* ≤ 0.002. (**D**) Immunofluorescence staining of LG epithelium specific markers (C-Kit, Lysozyme, Pan CK^7^) in human LG tissue and LG-Stromal cells. * In images indicates blood vessels in the LG tissue sections.

Upon immunostaining, LG-stromal cells and BM-MSCs revealed positive expression for panel of MSC markers like CD73, CD90, CD105, and Vimentin, and negative for CD45 and HLA-DR (Fig. 5A). Representative graph for total fluorescence intensity and total number of positive cells revealed similar pattern (Figs. 5B, 5C). Acinar cells in lacrimal gland tissues stained positive for C-kit, Lysozyme, and Pan-CK. Ki-67 positive proliferating cells were observed in LG-stromal cell population. However, LG tissues as well LG-stromal cells stained positive for ABCB5 (Fig. 5D).

### Trilineage Differentiation

**Figure 6.**
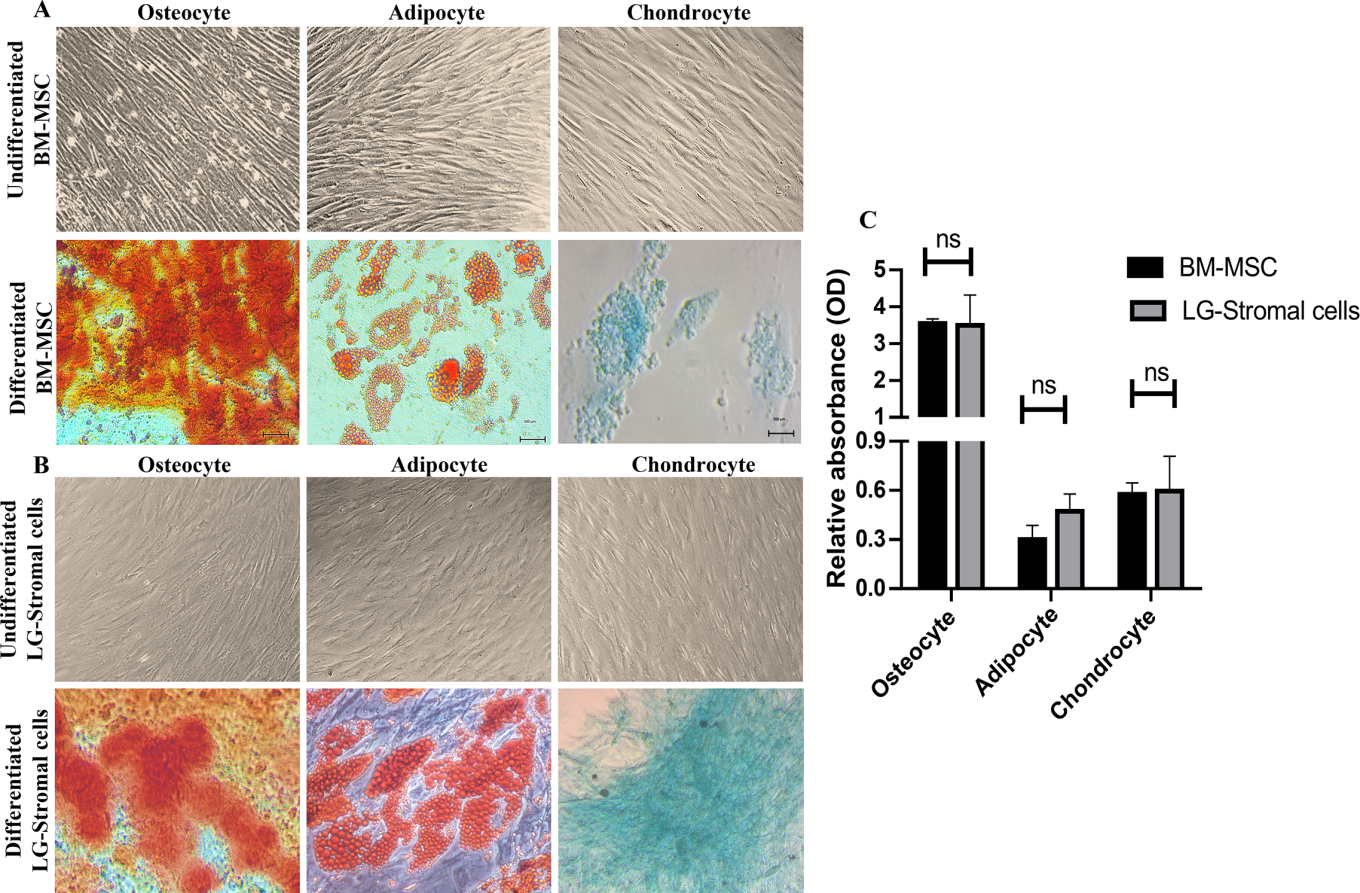
Characterization of primary BM-MSCs and LG-Stromal cells by differentiation assays. (**A, B**) Primary BM-MSCs and LG-stromal cells were cultured in osteogenic, adipogenic medium, and chondrogenic medium for 21 days, respectively. Osteocyte differentiation was confirmed by the presence of calcium deposits stained positive for Alizarin Red Stain. Adipocyte differentiation is evident in the presence of Oil Red-positive lipid-laden cells. Chondrocytes stained positive for Alcian blue stain. Undifferentiated cells (negative control) refer to the cells grown in complete DMEM F12 media. All images were captured at 40X. (**C**) Quantitative analysis of the differentiation capability of BM-MSCs and LG-stromal cells. Data are shown as absorbance mean ± standard deviation (*n* = 3).

Following 3 weeks of differentiation in specific media, LG-stromal cells and BM-MSCs grown in osteocyte induction media showed visible brownish black calcium deposits in the culture plates. These cells stained positive with alizarin red. Cells grown in adipocyte differentiation media developed round spherical oil/lipid droplets within the cells in culture plates. Adipogenic induction was confirmed by Oil Red O staining which showed lipid laden adipocyte phenotype of cells. Post differentiation cells grown in chondrocyte induction media stained positive for Alcian blue stain indicating chondrocyte differentiation. Both LG-stromal cells and BM-MSCs showed similar differentiation phenotypes upon induction. Representative images and quantification graphs shown in Figures 6A, 6B, and 6C.

### Gene Expression Analysis

**Figure 7.**
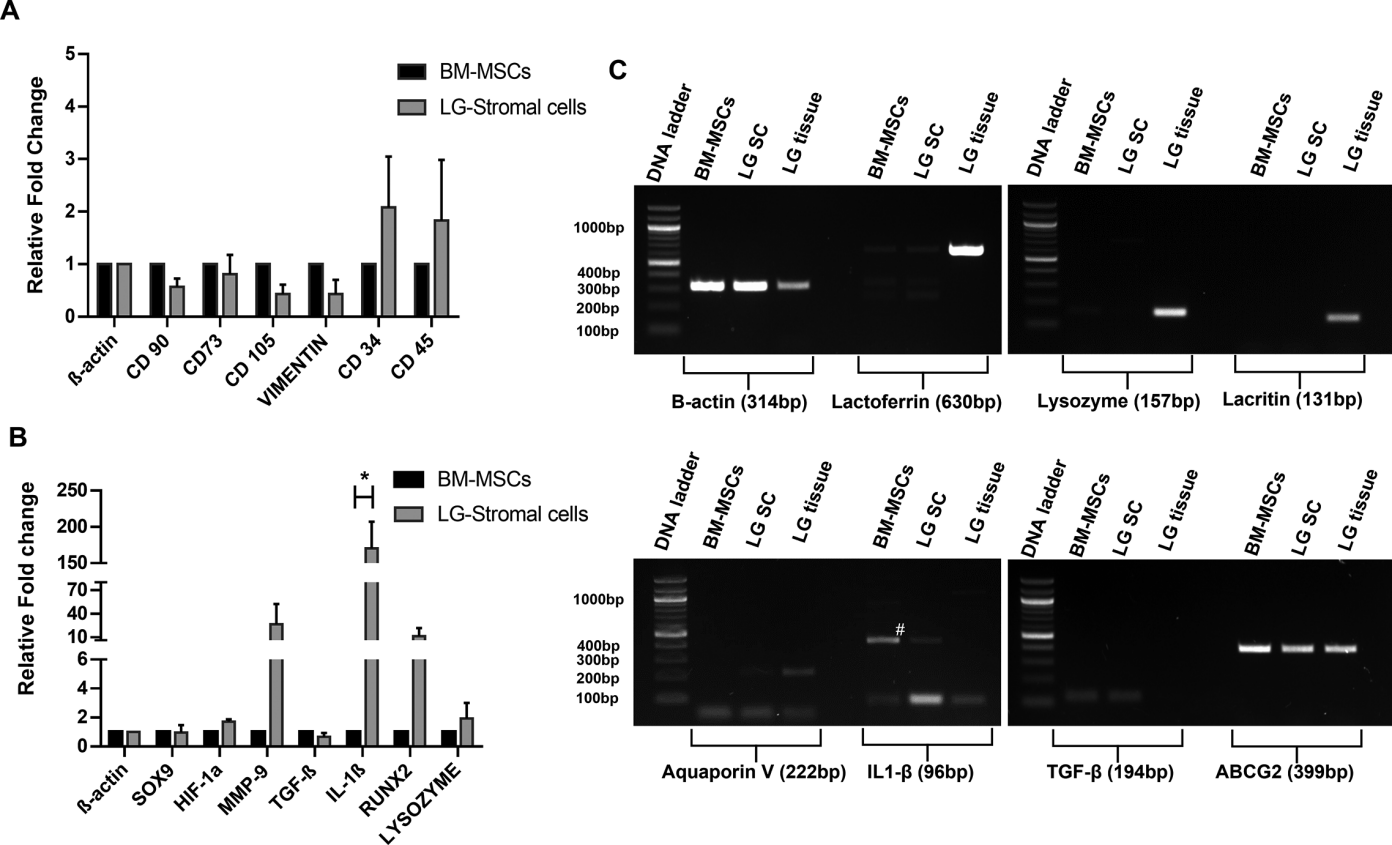
Gene expression of various markers plotted as their relative fold change values. The relative fold change values were calculated from the Ct values of LG-stromal cells with respect to BM-MSCs (normalized to 1). (**A**) The gene expression of MSC specific markers in BM-MSCs and LG-stromal cells. (**B**) The gene expression of other markers in BM-MSCs and LG-stromal cells (* *P* ≤ 0.0085). (**C**) Reverse transcription polymerase chain reaction analysis for the expression profiles of selected LG specific markers in BM-MSCs, LG-stromal cells (LG-SC) and the whole LG tissue. The 100 bp DNA ladder was used in gel images. A “#” in the gel image indicates the nonspecific band of IL1-β around 450 bp.

RT–PCR analysis of BM-MSCs and LG-stromal cells showed similarities in expression profiles of various MSC specific markers (Fig. 7A). There was no significant difference in the expression of other selected markers except for IL1β (P* ≤ 0.0085; Fig. 7B). The expression of LG epithelial specific markers, like Lacritin, Lysozyme, Lactoferrin, and Aquaporin V, were found uniquely in LG tissues, whereas the LG-stromal cells lacked expression of these markers. ABCG2 was abundantly expressed in BM-MSCs, LG-stromal cells, and LG tissues. However, TGF-β expression was observed in both the cell types whereas it was absent in LG tissue (Fig. 7C).

## Discussion

The LG is predominantly made up of epithelial cells, like acini, ducts, myoepithelial cells, and cultures from the primary LG tissues consists of all the cell types, that is, epithelial, mesenchymal, and myoepithelial cells.^7^^,^^8^ The presence of stromal cells in the cultures prompted us to explore their similarities with MSCs found in bone marrow and many other parts of the body, including the limbus of the eyes.^23^^,^^30^^,^^31^ The human LG tissues used in this study were procured from normal individuals undergoing LG debulking surgery and these individuals were non-diabetic and had normal peripheral blood parameters when checked for fitness for local anesthesia (i.e. no signs of systemic inflammation). Thus, the chances of MSCs from other sources to migrate and integrate the LG is minimal here, as it occurs in context of inflammation or trauma in a specific organ.^38^ Hence, we speculate that these are inherent cells of human LGs and are not migrated from any other source.

Although the murine mesenchymal cells have indicated a therapeutic role in DED, the evidence for the presence of MSC population in human LGs is not yet proven. In this study, we documented that the stromal cells obtained from human LG cultures show distinct similarities with bone marrow derived MSCs and hence could be considered as LG derived MSCs (LG-MSCs).

Some previous studies have indicated the presence of MSCs in murine LGs,^10^^,^^12^^,^^13^ and these cells were shown to have effects in alleviating the symptoms of DED. Even though MSCs from murine LGs were previously reported,^10^ the characterization of these cells based on the ISCT criteria and an established MSC control is still lacking. Nestin positive spindle shaped cells capable of differentiating into adipocytes were characterized as murine LG-MSCs.^10^ Similarly in our study, the cultured human LG-stromal cells also express Nestin and ABCG2, suggesting these are stem cells (Supplementary Fig. S1). Additionally, other studies in murine LGs have shown increased number of MSCs in regenerating LG owing to its therapeutic role in DED and LG regeneration.^12^ However, MSCs from other sources have been used in DED models and have shown its safety and efficacy upon transplantation.^39^^–^^41^

This study aimed at isolating and characterizing the stromal cells using the minimal criteria set by ISCT; these cells should fulfil specific conditions namely plastic adherence, spindle shaped morphology, and tri-lineage differentiation, and must express cell surface markers CD73, CD90, and CD105, and lack the expression of CD45, CD34, CD14, CD11b, CD79α, or CD19 and HLA-DR surface molecules.^15^ Our LG cultures initially showed mixed population of cells growing from the explants (see Fig. 1A), followed by more enriched stromal cells in further passages. Previous studies have reported usage of matrigel, human amniotic membrane (HAM) and collagen coating along with specialized (HepatoSTIM) media using high concentration of epidermal growth factor (EGF) as the standard growth media for culturing LG epithelial cells.^7^ Most of the MSC cultures have been using DMEM with 10% FBS as the basic media for their growth and propagation. The mice LG-MSCs were reported to be cultured in α-MEM, 2 mM L-glutamine, 15% FBS-S, and 1% penicillin/streptomycin.^12^ The MSCs from human limbus were grown in DMEM/F12 media fortified with 2% FBS, 1% [vol/vol] antibiotic-antimycotic, 10 ng/mL epidermal growth factor and 5 µg/mL insulin.^42^ In our study, the culture conditions for the growth of LG-MSCs from human LG were modified based on the previous literature for the growth and propagation of MSCs from various sources. Briefly, DMEM F12 with 10% FBS, 2 mM L-glutamine, 1% antibiotic-antimycotic, 10 ng/mL EGF, and 5 µg/mL insulin supported the growth of LG MSCs rather than the epithelial cells. By passage 3, pure population of LG-MSCs were isolated using these culture conditions (see Fig. 1). The human LG-stromal cells cultured from donors of various age groups and gender revealed similar cell morphology (Supplementary Fig. S2). The H&E staining of human LGs showed proper acinar structures with no inflammation and damage within the tissue (see Fig. 2A). In addition, the expression of MSC markers Vimentin and CD105 in human LGs revealed that these cells are seen surrounding the acinar structures of the LG (see Fig. 2B).

To understand the cell proliferation and clonogenic potential of these cells, colony forming efficiency (CFE; Figs. 3A, 3B) and population doubling time (PDT; Figs. 3C, 3D) were performed. Similar to the adherent spindle cells of BM-MSCs, LG stromal cells also formed small colonies (CFUs) when seeded at lower cell density. We have observed CFE of 16.13 ± 0.61% for BM-MSCs and 15.0 ± 1.0% for LG-stromal cells at passage 3 suggesting these cells are capable of growing into colonies from single adherent cells. With the increase in passage number, the PDT increased in both BM-MSCs and LG-stromal cells. These data were in coherence with other reports that showed the properties of MSCs.^23^^,^^31^ Nevertheless, increased proliferation rate of MSCs allows for shorter culture time and large expansion generating a sufficient population of MSCs which would be beneficial for clinical use.

The phenotype of LG-stromal cells showed remarkable resemblance to that of BM-MSCs, that is, they demonstrated positive expression for CD73, CD90, and CD105, and negative for CD34, CD45, and HLA-DR fulfilling the criteria of MSCs (see Fig. 4, Fig. 5A).^15^ Even though these cells show similar expression patterns, there was a significant difference in the total fluorescence intensity among LG-stromal cells and BM-MSCs (see Fig. 5B). However, we speculate that the higher fluorescence intensity in BM-MSCs could also be attributed to its larger size compared to LG-stromal cells. Upon staining of LG tissues, acinar epithelial cells in the lacrimal gland were positive for epithelial markers C-kit, Pan-CK, and lysozyme, which is an acinar secretion (see Fig. 5D). However, the LG-stromal cells did not express any of the acinar epithelial markers confirming these cells are not of epithelial origin. The LG stromal cells also demonstrated differentiation into mesodermal lineages-osteocytes, adipocytes and chondrocytes similar to BM-MSCs (see Fig. 6).^16^^,^^23^^,^^31^ We have further performed quantitative analysis to substantiate the data and no significant difference was observed between BM-MSCs and LG-stromal cells in their differentiation ability. Therefore, it can be concluded that the spindle shaped cells isolated from primary human LG cultures exhibit the characteristics of multipotent stem cells. However, the gene expression of the differentiated cells was not analyzed in this study.

Gene expression profile of LG-stromal cells and BM-MSCs have shown to confirm similar expression of MSC specific markers with no significant difference in the expression of CD90, CD105, CD73, CD45, CD34, and Vimentin (see Fig. 7A). However, the expression of some markers, like MMP9, RUNX2, and IL1-β, were found to have difference among both the populations (see Fig. 7B). Extracellular matrix remodeling is an outstanding property of MSCs which is found to be comparatively higher in LG-stromal cells by the expression of MMP9. Early activation of MMP9 is known to accelerate wound healing and hence in tissue remodeling.^43^ RUNX2 is one of the earliest marker gene for bone formation, and marks the differentiation into osteoblasts. It activates the transcription and expression of both Col I genes and the bone sialoprotein (BSP), the gene expression of RUNX2 was observed in LG-stromal cells higher than BM-MSCs, confirming that the stromal cells indeed promote a long-lasting expression of RUNX2. Significant increase in the expression of IL-1β was observed in LG-stromal cells compared to BM-MSCs. IL-1β is known to have important role in promoting angiogenesis^44^ and it could play a crucial role in the LG development and regeneration. Most of the studies use IL-1β to prime the MSCs from various sources prior to its therapeutic application,^45^ so that the downstream pathways are activated. Lysozyme is an important tear protein with antimicrobial properties and have protective roles in the ocular surface.^46^ LG-stromal cells do not express lysozyme unlike the epithelial cells of LG. We observed expression of Lysozyme, Lactoferrin, Lacritin, and Aquaporin V in LG tissues, whereas it is completely absent in the LG-stromal cells (see Fig. 7C).

We thus speculate that the LG-stromal cells are specialized cells of mesenchymal origin which might play a role in providing a healthy microenvironment in normal LG function or in repair of diseased LG in patients. However, the role of these cells in normal and disease conditions could not be extrapolated in this study. Further functional studies using human LG derived MSCs are required to prove its therapeutic role in DED and to understand its underlying molecular mechanisms.

## Conclusion

Our data show that the spindle shaped cells obtained from human LG cultures are specialized cells of mesenchymal origin with respect to their phenotype, culture characteristics, trilineage differentiation, and expression, and hence could be serving as LG tissue specific MSCs. Similar to the role of MSCs in other organs, we speculate that the LG derived MSCs could contribute to regeneration, and generation of 2D-3D LG organoids both in vitro and in vivo conditions, thus warranting further studies for validation.

## Supplementary Material

Supplement 1
