# Supplementary material for: Human Lacrimal Gland Derived Mesenchymal Stem Cells – Isolation, Propagation, and Characterization
Source: Invest Ophthalmol Vis Sci. 2023 Jul 13;64(10):12. doi: 10.1167/iovs.64.10.12 (PMC10353750; doi:10.1167/iovs.64.10.12)
Supplement: Supplement 1 [file iovs-64-10-12_s001.pdf]

Supplementary data:

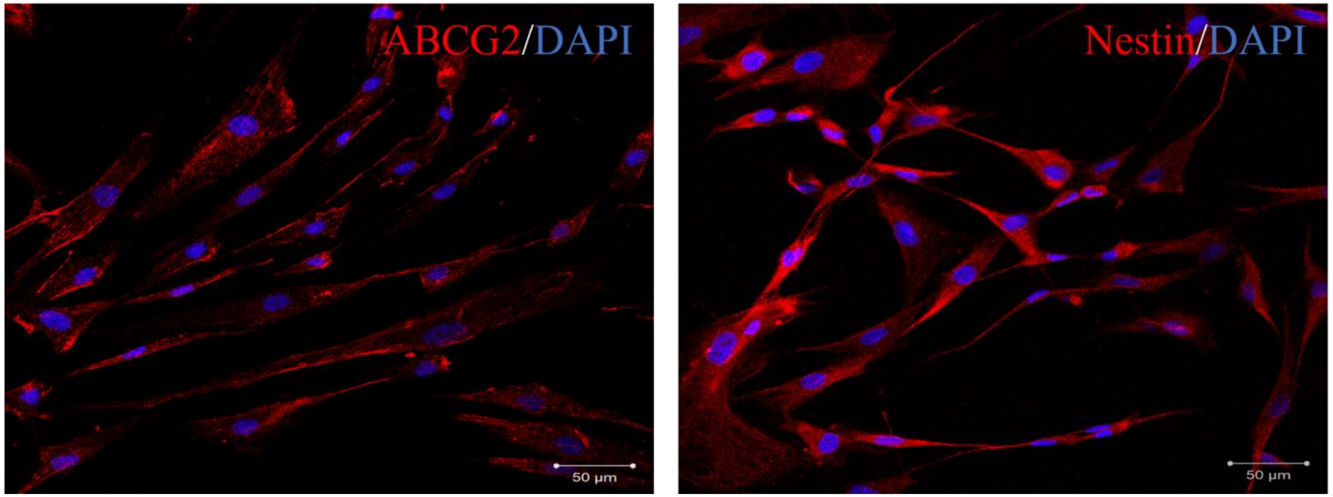

S1. Immunostaining of LG-Stromal cells:

Supplementary Figure S1. *The immunostaining of LG-Stromal cells shows expression of stem cell markers ABCG2 and Nestin. The nucleus was counterstained with DAPI. Images were captured at 20X magnification.*

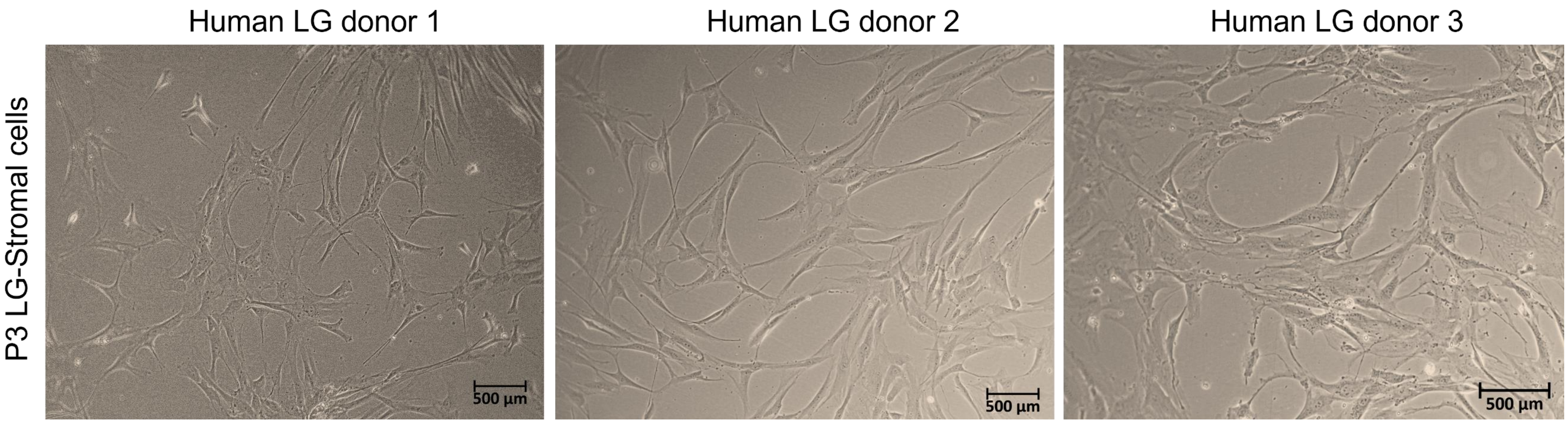

S2. Human LG-Stromal cell culture from multiple donors:

Supplementary Figure S2. *The culture of human LG-Stromal cells from three different donors shows similar spindle shaped morphology of cells at Passage 3. All images were captured at 10X magnification (Human LG donor 1- 11/M, LG donor 2 - 40/M, LG donor 3 - 64/F).*

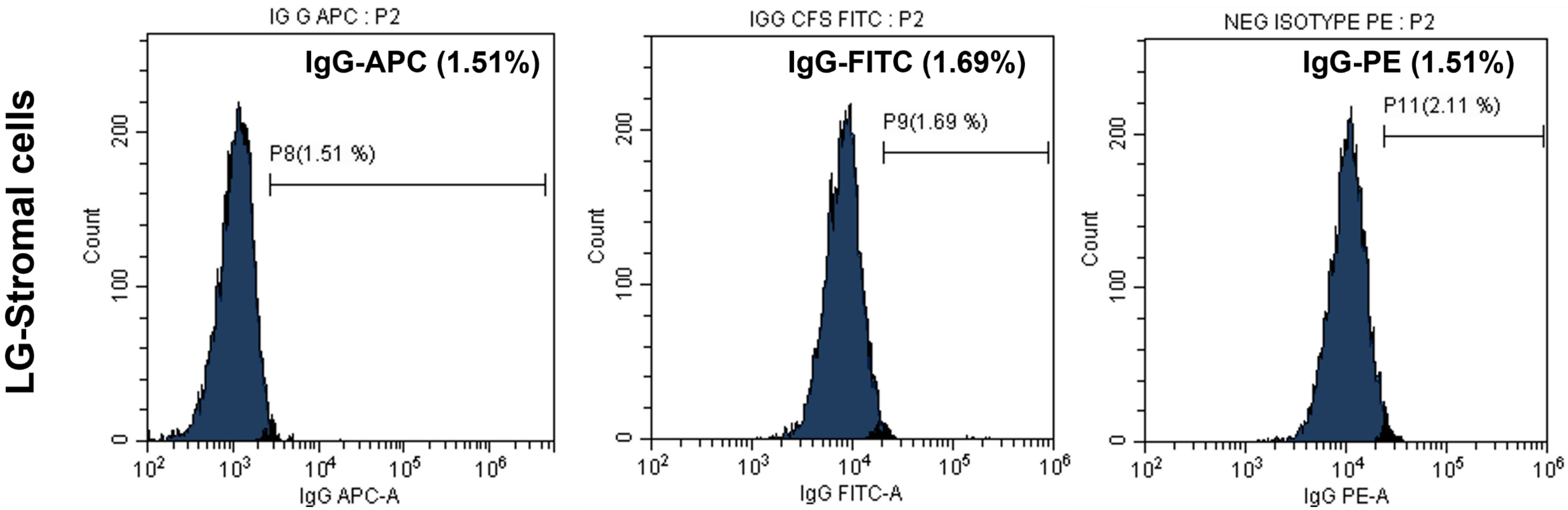

Supplementary Figure S3. *The isotype controls used in the flow cytometric analysis of LG-Stromal cells show <2% expression.*
